# Fermenting Alvine Excretions as a Source of Disease

**Published:** 1856-07

**Authors:** C. H. F. Routh

**Affiliations:** Physician to the St. Pancras Royal General Dispensary, Assistant-Physician to the Samaritan Hospital, and Vice-President of the Medical Society of London.


### ON FERMENTING ALVINE EXCRETIONS AS A SOURCE OF DISEASE.

By C. H. F. ROUTH, M.D., Physician to the St. Pancras Royal General Dispensary, Assistant-Physician to the Samaritan Hospital, and Vice-President of the Medical Society of London.

HUMAN alvine dejections, according to Dr. Marcet's analysis, consist of *excretine,* a peculiar animal matter; *margaric acid,* an oily acid; *colouring matter,* like that of the blood; a light *granular substance,* which is believed to be a combination of phosphate of potash and a pure organic matter; and an olive-coloured acid, *excretoleic acid.* There is no evidence of butyric acid. Excretine contains both sulphur and nitrogen, and is not found in the excreta of the carnivora. But a substance like it, together with butyric acid and cholesterine, exists in those animals. In the herbivora, neither *butyric acid, excretine,* nor *cholesterine* is found, except in the crocodile, in which cholesterine exists. The salts of these matters are, in the main, potash, lime, phosphoric acid, and magnesia. Human dejections are richer in nitrogen than those of other animals; they yield 4 per cent. to the latter 2 per cent., at least if the food be animal and not vegetable in excess; 7 per cent. consist of undigested residue of food. A very small quantity of this excretion can be looked upon as secretion in health, although in disease some matters may be abnormally secreted, as in cases of colliquative diarrhœa in consumption, or of excess of the alvine dejection where the same amount of food is taken: gaseous products are excreted in tympanitis, or when two ends of an intestine are artificially tied. Lastly, excretive matter can be artificially prepared out of the body by the action of certain acids on the ordinary products of food.

When the bowels are unusually constipated in certain cases, the matters retained undergo a species of *fermentation,* which may be of two kinds: *eremacausis, i.e.,* changes effected by exposure to moisture or air at certain temperatures, but without production of odour; and, secondly, *putrefaction, i.e.,* the same with offensive odour. In regard to the first of these, many of the normal healthy changes in the body are products of fermentation; and all animal substances, such as flesh, blood, bile, urine, the mucous membrane of the stomach, intestines, or bladder, or the skin, may act, if putrescent, as ferments, each giving rise to a particular kind of fermentation, which will go on so long as there is a substance present from which the ferment was originally formed, and whenever there is a compound capable of being decomposed by contact by the ferment.

There are certain circumstances that favour putrefaction, and others that prevent or retard it. First, oxygen favours it, especially when diluted with nitrogen. Hypophosphoric acid has the same effect; pure nitrogen retards it; hydrogen, carbonic acid, and nitrous acid also retard it. Electricity favours it; if meat, however, be put in an electro-negative state, it repels oxygen, and so keeps fresh for a long time. Aqueous vapour favours it; very dry air retards it. A stream of water delays the change: also the water of privies—this being due to the large excess of ammonia present. Creasote and smoke arrest or prevent it. Dr. Richardson finds that nitrogen gas and chloroform vapour, or the presence of phosphorus, prevent putrefaction. Sea water, especially if diluted with fresh water, favours decomposition.

With regard to fermentation in the body, it seems, from some experiments by Magendie and Chevreuil, that carbonic acid, nitrogen, hydrogen, carburetted hydrogen, oxygen, and sulphuretted hydrogen, are formed in the intestines during digestion, by fermentation; this fermentation, however, is greatest in the large intestine, because unimpeded by the presence of free acid. Dr. Ayres, in addition to these gases, mentions phosphuretted hydrogen. Sometimes, however, this fermentation proceeds to putrefaction, as shown by Lehmann and Frerichs, who found in retained and putrefied alvine dejections substances precisely similar to those obtained by Bopp from putrefying protein bodies. Dr. Ayres has indicated some of the changes which occur in putrefying night-soil, from which ammonia, sulphuretted hydrogen, and a very fetid liquid can be obtained. In common sewers two kinds of gases are evolved; one common, which consists of oxygen, 13; nitrogen, 81; carbonic acid, 2; sulphuretted hydrogen, 3; besides a small quantity of ammonia: another less common, and consisting of oxygen, 2; carbonic acid, 4; nitrogen, 94; sometimes with ammonia. Alvine dejections sometimes undergo remarkable changes in the body even during life, not to speak of the admixture with these of blood, pus, etc. They vary in chemical reaction, sometimes being very acid, sometimes neutral.

*Infection through alvine matters* occurs in three ways: I. When such matters are absorbed in their natural concentrated state. II. In solution, or after suspension in water. III. When the emanations therefrom are inspired. But, in order that infection should be propagated, three conditions are required: 1. An infecting source. 2. A transmitting medium. 3. A fit recipient.

1. The infecting source may be the sick person, his fomites, excreta, etc. These sources are always more concentrated and potent at the beginning of an epidemic, weaker at the end.

2. The transmitting medium may be (*a*) *liquid*, as serum or blood, as in inoculation; or water in which poison is suspended or dissolved, and taken into the body; or it may be (*b*) *gaseous*—air containing a volatile substance. The thermometrical and barometrical changes which influence the gaseous infection may be the reverse of those which influence the liquid; the gaseous being more energetic in *hot* or *moist* weather, the liquid in *temperate* and *dry* weather; because in the first case the air is lighter, and the ammonia (which helps to suspend volatile poisons in the air, and to raise them to the nose) exists in larger quantity. Hence the foul smells of drains are more sensibly experienced at such periods; whereas in moderately cold and dry weather the air is heavier, less ammonia is generated, and the poisons are kept nearer the surface of the earth.

3. There must be fit recipients. The fitness, as such, will be modified by *race*, as in the case of a negro, who is less susceptible to fever than a white man; by *species* in animals, as is illustrated in the fact that the dog is innocuous to inoculation with plague matter; by *concentration* of the poison, which will infect the same man by its greater potency at the beginning of an epidemic rather than at the end; by *acclimatisation*, the effects of which are seen in a yellow fever district, where the old residents suffer less than new comers; by *previous disease*, as instanced in the well known fact that a disease may sometimes not be taken more than once; by *occupation*, which renders some men more exposed than others; by *state of health*, and by *food* supplied. These are all modifying causes; and, if they are not duly attended to, the greatest confusion results in the observation of an epidemic.

I. Effects of Alvine Dejections in Substance. Healthy dejections are not poisonous; those of the horse, for instance, formed as they are from retained nutritive matters, are eaten with impunity by birds. The experiment is also voluntarily performed by voracious dogs, and even by hysterical and demented human beings, with impunity. Diseased alvine dejections are, however, poisonous; that is, those in which fermentation has occurred. The simple retention of these in the bowels may give rise to serious disease, and aggravate all forms of epidemics. Such are the opinions of Burn, in his work on *Habitual Constipation*, and of Hamilton, in his work on *Purgatives*. When matters have been long retained in the bowels they give rise, on the one hand, in extreme cases, to symptoms very analogous to those produced by the inoculation of putrid matters in the blood, viz., *low fever and typhoid symptoms*, and *delirium;* or, on the other hand, to those symptoms produced occasionally when putrid animal matters, as sausages, cheese, and pork, are partaken of—symptoms, I mean, of *violent purging and vomiting*, in more acute cases, and of dyspepsia, general wasting, debility, nervous affection, in cases where the poison acts more gradually. The habitual foul breath and tongue, and the generally offensive odour of persons who are habitually constipated, is evidence of the same slow poison in action. The very propinquity of alvine matters causes the pus in abscesses in the neighbourhood of the bowels to undergo putrefaction. The effects of cholera dejections on man and the lower animals are peculiar. Five case have been quoted by Dr. Richardson, in his review on the water supply in London (JOURNAL OF PUBLIC HEALTH, vol. i, p. 130), in which five persons partook of these; four took cholera, and one died. The experiments upon animals are unsatisfactory. Those made by Thiersch with putrid choleraic dejections, communicated cholera to white mice; but in other hands, although violent purging and vomiting in dogs and cats were induced, and death occasionally occurred, the algide symptoms of cholera were absent. There is reason to believe that typhus and yellow fever are occasionally produced by the retention of putrid alvine matters. One variety of malignant puerperal fever, accompanied with mania, was without doubt produced by a similar cause; and there are ordinary cases of mania, which may possibly be traced to the same poison in operation.

II. FERMENTING ALVINE DEJECTIONS AS A CAUSE OF DISEASE, AFTER SOLUTION OR SUSPENSION IN WATER. Some substances require a certain amount of *moisture* to ferment, *i.e.* to absorb oxygen; thus meat in dry oxygen will keep for a long time, but will be speedily decomposed in moist. If a *small amount of ferment is added* to a body in water, the body decomposes much sooner than if it were left to generate independently a ferment. Water under certain circumstances contains an excess of oxygen. Some animalcules, such as *glamidonas pulviculus*, generate oxygen in large quantities; and several water plants, such as the *valisneriæ* and *anacharis*, emit oxygen gas. Water under these circumstances highly favours fermentation. Perhaps also this process is encouraged by an excess of chemical rays in the light. In many cold countries there is excess of oxygen in a given volume of air, from the increased density of the atmosphere. This excess is also found in snow; hence, if snow is soiled by slops thrown upon it, and is subsequently melted down for drinking water, and so contaminated is partaken of, the matters contained in it soon begin to ferment in the alimentary canal; a fact which helps to explain one cause at least of cholera in St. Petersburg and Moscow, when the temperatures were below zero, for emanations at such temperatures are impossible. But a dry and dense atmosphere is not necessarily confined to, though more common in, cold weather. Experience proves that during some epidemics the air is both dry and heavy, while the water is abnormally warm. This was the case in the three epidemics of cholera of 1832, 1848, and 1854; the air being also in a weak, positive electrical state—a state, as before seen, favourable to putrefaction.

Fermenting alvine dejections may be productive of particular diseases.

1. *Cholera.* Three epidemics of cholera,—one in Newcastle, another in Lambeth, and a third in Golden-square,—all afford, as set forth by Dr. Snow in his work on Cholera, indications of the propagation of disease by fæcal matter.*

*a. Newcastle and Gateshead.* The deaths in Newcastle in 10,000 population, were in 1831, 182; in 1849, 41; in 1853, 178. In Gateshead, in 1849, there was a comparative immunity; in 1853, 166 deaths occurred. In 1831, there were no waterworks; in 1849, the water supplied was good; in 1853, it was bad. The waterworks in both towns are the same. Before 1832, the water was obtained from the Tyne, a mile above the town, the tide flowing six miles beyond it. In 1848, waterworks were made, and water was supplied from Whittle Dean, a stream ten miles above the town. In 1853, the quantity supplied from this source was found insufficient, and the original waterworks of 1832 were again had recourse to. This water was so bad in 1853, as to contain 15.6 per cent. of impurities. As the water supplied was pure or impure, so the mortality from cholera was low or high. It was greatest from the 13th to 23rd of September. Owing to the outcry against the Company, no more Tyne water was supplied after the 15th, and the Tyne water was out of the pipes about the 17th, from which time the mortality decreased. As corroborative evidence, it may be remarked, that the inhabitants of the places supplied with *pump water*, and not with that of the Company, suffered only from diarrhœa, not from cholera.

---

* See also Journal of Public Health, vol. i, p. 130.

*b*. The *Lambeth district* of London is supplied with water from two sources: the Lambeth Company's works, and those of the Southwark and Vauxhall Company. The Lambeth Company, in 1849, got its water from the Thames near Hungerford; in 1854, it obtained it from Thames Ditton. The Southwark and Vauxhall Company has always obtained its water supply from near Battersea Bridge. In 1849, the mortality was pretty near the same in both districts. In 1854, the mortality of the district supplied by the Southwark and Vauxhall Company was, for the first four weeks, twenty times as great as in the district supplied by the Lambeth; and for the next three weeks, eight times as great. The cause of this difference is due to the fact that during the period between the two epidemics, the source of supply for the Lambeth Company's works had been changed,—being taken many miles higher in the Thames. It was thus less impregnated with the London sewage than was the water of the Southwark and Vauxhall Company, which, on examination, was found to contain alvine matters in suspension.

*c*. *Golden-square*. Upwards of five hundred deaths occurred here in ten days. The outbreak commenced on the night between the 31st of August and 1st of September, and was distinctly traced to the drinking of water from a particular pump in Broad-street. The history of the cases has been most ably traced out by Dr. Snow. Persons who partook of the water of the Broad-street pump, though they lived at a distance, took cholera and died. Those who lived near it, and partook of other water, escaped. It was found subsequently that in a house opposite the pump, on a day or two preceding the outbreak, a boy had had cholera, and that the pipe of the water-closet conducting from this house passed close to the pump pipe; and, the mortar work between the two pipes being broken down, the alvine dejections of this cholera patient were thus freely intermixed with the pump water. A comparison was then made between the water of the water companies and of pumps. Both contained organic matter, dead and living; annelidæ, dromelaceæ, diatomaceæ, infusoria, confervæ, fungi, hairs of animals, starchy matter, vibriones, but no animal peculiar to cholera. The water of the Southwark and Vauxhall, Lambeth, and West Middlesex Companies, contained three specimens of sea-water animalcules. A few of the pump waters contained a notable quantity of sewage matter. The waters from very deep wells were remarkably free from organic matter.

2. *Typhus and Typhoid Fever*. In an epidemic of typhus fever which broke out in Hastings, the disease was traced to bad water, the dejections of a typhus patient having been mingled with the system. The Croydon typhoid fever of 1852, and an epidemic which broke out in North Boston, Erie County, U.S., were traceable to the same general cause. In many parts of London, owing to the nature of the soil, more or less sewage matter becomes mixed with the water. The absence of smell in such waters or its clearness is no proof of its being uncontaminated. Generally speaking, there is typhus fever present wherever the water is thus poisoned.

3. *Diarrhœa* is a common effect of drinking bad water. Some very bad kinds of dysentery are also produced by this cause, and instances are plentiful where the disease has broken out in consequence of an admixture of sewage matter with drinking water. The same cause was in operation in the production of the plague at Cairo; possibly it is so in some epidemics of yellow fever, as shewn by Dr. Snow.

The effect of fermenting poisons, after their development in water, seems to be more powerful than when they are taken in substance; and to be somewhat modified both in degree and kind, as we shall hereafter see, when they are inhaled as emanations.

III. Emanations from Alvine Dejections as a Poison. There are three admitted conditions necessary for the emanation of miasmata; 1st. A certain amount of temperature; 2nd. Moisture in the air; 3rd. The evolution of ammonia. An opposite state leads to deposition on the surface or in water. Emanations are not possible below 32 F. The ammonia, if formed then at all, will keep to the surface; and the same is true of watery vapour. A cubic inch of air at zero F. can only contain .836 grain of moisture, while at 95 F. it contains 17.009 grs. At 11 F. almost .0 grains are contained, while at 50 there are 2½. In dry fine weather, especially if cold, drains do not smell; in damp weather they are very offensive. So true is it that dampness often exists with epidemics, that Dr. Barton, of the United States, has founded thereon a theory, which has been supported by Dr. Hunt of Buffalo (*Transactions of the American Association*, 1854), that without the conjunction of dampness and what he called terrene causes, no epidemic can occur. Terrene causes are any causes which may give rise to miasmata, such as upheaval of soil, and decay of organic matter. But, besides watery vapour and ammonia, there are probably other gases evolved, such as carburetted hydrogen, sulphuretted hydrogen, phosphuretted hydrogen, and carbonic acid. In the decomposition of alvine matters there are the two kinds of compound gases before alluded to; the second of which kills because it is irrespirable; the first, because it contains an excess of sulphuretted hydrogen. The symptoms produced by phosphuretted hydrogen and carburetted hydrogen are not clearly made out, particularly when small quantities only are inspired. The symptoms produced by the inspiration of sulphuretted hydrogen in small quantities are those of gradual prostration of the physical powers, general debility, followed by emaciation, and low fever; generally, too, all diseases, if long exposed to sulphuretted hydrogen emanations, assume a typhoid and putrid character. The temperature at which these diseases develope themselves varies probably with each disease; however, the ferment is destroyed at very high as well as at very low temperatures. Yellow fever ranges between the temperatures of 60 and 90. But there can be no doubt that something more is required, and this is to be found in the *electrical* state of the atmosphere. We have already seen that a positive electrical state of the atmosphere favours decomposition, and therefore disease in man. The same state of electricity favours vegetation: hence, perhaps, the concurrence of very active vegetation in pestiferous districts. As vapours ascend, so does positive electricity. This effect is less powerful in dry air. In some cases the electricity of the animal body is reversed. In health the mucous membrane gives negative electricity, and the skin positive: in typhus, these conditions are reversed.

*Injurious Influence of Night Soil Emanations.* Drs. Lewis and Sutherland give instances of this. Near Christ Church workhouse, Spitalfields, there was, in 1848, a manufactory for artificial manure; this caused a most powerful stench. Whenever the wind blew in that direction, then numerous cases of fever of typhoid character, a typhoid tendency to measles, small-pox, and other typhoid diseases, and an intractable and fatal form of aphthæ, originated. In December 1848, sixty of the children were seized with diarrhœa in one morning. The manufactory was obliged to cease its operations, and so disease ceased in the workhouse. Five months afterwards, it was reopened. The night following, the direction of the wind being towards the workhouse, forty boys were seized with diarrhœa; the girls, whose dormitories faced in another direction, escaped. The nuisance was again suppressed, and the disease ceased. The propinquity of a similar manufactory to St. George's, Southwark, produced similar results, and were removed by the same measure. An epidemic of cholera broke out amongst the inhabitants of Crafton-terrace, Latimer-road, concurrently after the wind had blown towards it from the potteries at Kensington, a locality giving rise to most offensive odours from excess about it of putrefying night-soil and animal matter. The suburb of Witham, in Hull, a space of some three acres, on which night-soil and other manure is deposited, gives as the mean age of life 18, while in Hull it is 23. An apparent exception to the unhealthiness of such localities has been mentioned; viz., Montfaucon, in Paris, which is the great receptacle of the alvine matters of the Paris population. It is alleged that the health of the workmen engaged in that locality is excellent; typhus and low fevers seldom occur. The explanation is this. Good food is one of the best preservatives against such diseases. These workmen live on wholesome, although to us distasteful food; viz., horseflesh.* The other workmen of Paris have chiefly a vegetable diet. But, assuming that the locality itself is not insalubrious to the workmen, it is so at a distance, to the patients of St. Louis hospital, in the Faubourg St. Antoine, in which, whenever the wind blows from Montfaucon, puerperal fever breaks out among the lying-in women in that establishment.

Some of the diseases capable of being produced by emanations from excreta have now to be noticed.

1. *Cholera*. Pettenkofer believes that all diarrhœal fluids during choleraic periods will, after fermentation, develope choleraic poison; which, in its turn, will give rise, according to the case, to cholera, cholerine, or diarrhœa. At any rate, it has been proved by Drs. Alison and Budd, that the emanations from water-closets in which cholera dejections have been evacuated will produce cholera. The well marked case of a certain workhouse, in which all patients who were allowed to go to a contaminated water-closet caught cholera, while those who were prevented from going to it escaped, and this although both sets drank the same water, is evidence of this fact. The sufferings of our troops when made to encamp in the same place where the Russian troops had previously encamped and suffered from cholera, is a proof of a similar kind; although the effect may be in part explained by the bad water drank. The hypothesis is still more strikingly supported by the experiments of Dr. Lindsay, in which the exposure of animals to the emanations arising from the de-

* See JOURNAL OF PUBLIC HEALTH, vol i, p. 192.

jections and blood and clothes of cholera patients gave rise to the specific characters of the disease in four instances, and to death in two. The apparent contradictions afforded by other experiments with an opposite result, are to be explained by reference to the different sources of error before dwelt upon. The suburb of Witham, before alluded to, proved very obnoxious to cholera, ninety deaths occurring in a small space of two hundred yards. The mortality in the prison at Brest was also very great. This prison has four wards, besides an infirmary. The wards have twenty-seven water-closets, communicating with a drain which terminates in the harbour. The infirmary is differently constructed. At low water the south winds blow from the harbour up the drain, filling the wards with poisoned vapours. The number of cases of cholera in the wards was 6·7 per cent., while in the infirmary it was only 1·3 per cent. Of the eight deaths from cholera in Hampstead, four took place in localities infested by emanations from night-soil. In Marylebone those generally suffered most who lived over stables.

2. *Dysentery*. There can be no doubt that this disease, particularly in certain malignant forms, can be generated by emanations arising from alvine dejections. The writings of Hildanus, Pringle, Sennertus, Chomel, Zimmermann, and others, prove that the disease may be generated by this cause. A particular instance illustrating this fact occurred in Morovelho, where dysentery was maintained either by the alvine matters being kept in open pans placed under the beds in the hospital, by the same water-closets being frequented by all the patients, or by the slops being emptied into the same; but when measures were taken to remove the pans and keep the place sweet, the disease soon disappeared. Dr. James Bird observed the same result in the Indian hospitals during the first Punjaub expeditions. The emanations from the dejections reproduced dysentery in others, and moreover developed hospital gangrene in the hospitals. During this period the air was hot and moist. In the second Punjaub expedition, the disease did not spread; but then the air was cold and dry, proving the powerful concurrent effects of heat and moisture.

3. *Typhus and Typhoid Fever*. Allusion has already been made to the prevalence of these diseases in the case of the Christ Church Workhouse. The writings of Mr. Grainger show that the air of cesspools greatly predisposes to typhus. In Munich, typhus is a disease unusually prevalent; for two-thirds of the town are without drainage, and the night-soil is collected in large holes in the yards. Gaol fever, plague, and yellow fever are possibly developed, or are, at all events, very much intensified by the same agency.

*Rules of Treatment to be adopted.* If the views enunciated above are correct, the treatment to be followed is to give those remedies which impede or check fermentation; that is, *antiseptics*, such as the alkalies, the mineral acids, concentrated vegetable acids, volatile oils, alcohol, sulphurous acid, the metallic salts, mercury, arsenious acid, chlorine, creasote, phosphorus, charcoal, and tribasic phosphate of soda. Now, experience proves that these very remedies are those which have been found most useful in the treatment of these diseases, thus chalk mixture and alkaline earth have been given in diarrhœa; sulphuric acid and saline injections in cholera; mercury in fever; charcoal in dysentery, cholera, and fever. Further experiment will prove which antiseptics are most applicable to each disease. These measures, however, refer only to individuals. The several poisons arising from alvine dejections have to be combated in their origin and formation; for this object two agents have been recommended, *charcoal* and *fresh sea water*.

*Charcoal*, properly speaking, is not so much an antiseptic as a destructive; that is to say, it has the property of absorbing oxygen, together with the miasmata and the bad odour, and of consuming them by a process of low combustion. This subject has been extensively treated of by Dr. Stenhouse, and also in the JOURNAL OF PUBLIC HEALTH by Dr. Richardson. Charcoal filters, if placed over gully-holes and water-closets, would render essential service. Still, all sewers would continue to emit foul odours, and some gully-holes must be left open to allow the mud and the rain to pass down. It is manifest, therefore, that the best plan is to deodorise the sewers themselves. To do this, hydrochloric acid, chloride of lime, and pure charcoal, would be too expensive. Mr. Durden has suggested the preparation of charcoal by the action of sulphuric acid upon sawdust as the best, because the cheapest, mode of preparing it; but there would even be a difficulty in procuring sawdust on a large scale. The plans usually adopted for deodorising manure appear, perhaps, preferable. These are, first, Mr. Salmon's method, which consists in burning the slime and mud at the bottom of large rivers, particularly those which pass through large cities. The amount of animal and vegetable matter contained in this slime is very great, and when burned, it constitutes a very effective charcoal. Secondly, Mr. Alfred Sampson's method, which is simply to use the cinders of peat or turf, or the simple refuse of carbonised peat. Third, the bran obtained from sawn wood, the refuse of oak used for tanning purposes, the mould of the Parisian strata, or even simple clay, if carbonised, constitute an excellent disinfecting powder. Fourth, and lastly, Callond's method consists in the addition of the waste liquor of salt works to offensive matters, by which they are completely deodorised. It is stated by some, that carbonised peat is not so efficacious as other kinds of charcoal; if so it would appear that the method of Salmon is, after all, the most practicable, at least for London; at the same time, as the removal of the slime in and about the Thames would be of great benefit to the navigation of the river, and charcoal could be obtained from it in large quantities, and be mixed with the water used for flushing the drains, all odour would cease. All that would be required would be its forcible agitation with the water before it was thrown down in the drains. The same charcoal, used commonly in our water-closets, would remove all local bad odours; and the produce of the sewers would thus constitute ready made manure.

*Fresh Sea Water.* To say nothing of the expense of bringing sea water to London, this plan does not promise much; for, from experience at the sea-side, the inference is fair, that in warm weather, owing to the excess of sulphates, which would decompose, and liberate sulphuretted hydrogen in contact with animal and vegetable matters, the odours of our streets would be most offensive if watered with sea water. These sulphates, it should be remembered, amount to nearly four parts per thousand in the sea water in our channel; although in some seas (Mediterranean) they amount to seven parts per thousand, constituting, that is, from one-seventh to one-fourth the total quantity of the salts present in sea water. Moreover, sea water of itself contains a vast quantity of animal matter. The use of charcoal would be necessary to destroy this last, but it would not remove the sulphates. Hence bad Thames water, or the Serpentine water, would be preferable, if first purified by charcoal, to water our streets or to flush our drains.

In concluding this paper, I would state that many of the remarks made on the spread of epidemics, although applied especially to fermentation of alvine dejections, apply equally to diseases arising from the decomposition of other animal and vegetable matters. The same general principle obtains, in fact, in all, both as regards prophylaxis and treatment. I believe that much disease is produced and extended by the causes described, but that, with a little good will and industry, it might be entirely removed or prevented.

